# Development and validation of a simplified method to generate human microglia from pluripotent stem cells

**DOI:** 10.1186/s13024-018-0297-x

**Published:** 2018-12-22

**Authors:** Amanda McQuade, Morgan Coburn, Christina H. Tu, Jonathan Hasselmann, Hayk Davtyan, Mathew Blurton-Jones

**Affiliations:** 1 Department of Neurobiology & Behavior, University of California, 3014 Gross Hall, 845 Health Science Rd, Irvine, CA 92697-4545 USA; 2 Sue and Bill Gross Stem Cell Research Center, University of California, 3014 Gross Hall, 845 Health Science Rd, Irvine, CA 92697-4545 USA; 3 Institute for Memory Impairments and Neurological Disorders, University of California, 3014 Gross Hall, 845 Health Science Rd, Irvine, CA 92697-4545 USA

**Keywords:** Microglia, Neurodegeneration, GWAS, Stem cells, iPSCs, Hematopoietic precursor cells, Phagocytosis, TGFB, IDE1, Differentiation

## Abstract

**Background:**

Microglia, the principle immune cells of the brain, play important roles in neuronal development, homeostatic function and neurodegenerative disease. Recent genetic studies have further highlighted the importance of microglia in neurodegeneration with the identification of disease risk polymorphisms in many microglial genes. To better understand the role of these genes in microglial biology and disease, we, and others, have developed methods to differentiate microglia from human induced pluripotent stem cells (iPSCs). While the development of these methods has begun to enable important new studies of microglial biology, labs with little prior stem cell experience have sometimes found it challenging to adopt these complex protocols. Therefore, we have now developed a greatly simplified approach to generate large numbers of highly pure human microglia.

**Results:**

iPSCs are first differentiated toward a mesodermal, hematopoietic lineage using commercially available media. Highly pure populations of non-adherent CD43^+^ hematopoietic progenitors are then simply transferred to media that includes three key cytokines (M-CSF, IL-34, and TGFβ-1) that promote differentiation of homeostatic microglia. This updated approach avoids the prior requirement for hypoxic incubation, complex media formulation, FACS sorting, or co-culture, thereby significantly simplifying human microglial generation. To confirm that the resulting cells are equivalent to previously developed iPSC-microglia, we performed RNA-sequencing, functional testing, and transplantation studies. Our findings reveal that microglia generated via this simplified method are virtually identical to iPS-microglia produced via our previously published approach. To also determine whether a small molecule activator of TGFβ signaling (IDE1) can be used to replace recombinant TGFβ1, further reducing costs, we examined growth kinetics and the transcriptome of cells differentiated with IDE1. These data demonstrate that a microglial cell can indeed be produced using this alternative approach, although transcriptional differences do occur that should be considered.

**Conclusion:**

We anticipate that this new and greatly simplified protocol will enable many interested labs, including those with little prior stem cell or flow cytometry experience, to generate and study human iPS-microglia. By combining this method with other advances such as CRISPR-gene editing and xenotransplantation, the field will continue to improve our understanding of microglial biology and their important roles in human development, homeostasis, and disease.

**Electronic supplementary material:**

The online version of this article (10.1186/s13024-018-0297-x) contains supplementary material, which is available to authorized users.

## Background

Microglia are highly specialized tissue resident macrophages within the brain. Their homeostatic functions include shaping neural circuits through promotion of neuronal growth and differentiation as well as synaptic pruning. Microglia have also been strongly implicated in a number of neurological diseases and injuries. Most recently, genetic studies have identified many genes that are highly expressed in microglia which are associated with altered risk of developing Alzheimer’s disease (AD), Parkinson’s disease, Frontotemporal Dementia, or Amyolateral Sclerosis [1–4]. These new discoveries have placed microglia and neuroinflammation at the forefront of disease progression emphasizing the need for new model systems to enable the study of human microglia. Yet, microglia have proven to be difficult cells to study given that many differences exist between human and murine microglia [5]. Additionally, there are significant challenges in isolating and culturing these cells [6, 7]. Primary human microglia can be isolated in relatively limited numbers from postmortem brain tissue or following surgical resection of brain tumors or epileptic foci. However, given the considerable sensitivity of microglia to environmental changes, samples isolated from patients with neurological disease or following the agonal state prior to death, are likely to be activated and may differ depending on disease state, comorbidities, or cause of death. In order to study a more homeostatic human microglia and to utilize modern experimental manipulations such as CRISPR gene editing, many scientists have instead turned to induced pluripotent stem cells (iPSCs).

In the past 3 years, several labs, including our own, have developed various protocols for differentiating microglia-like cells from pluripotent stem cells [8–13]. While the purity, yield, and reproducibility of these different approaches varies considerably, each of these methods produces myeloid cells that exhibit transcriptional profiles and many key functional or morphological characteristics of human microglia. However, the relatively complex nature of these protocols has made it challenging for labs new to stem cell culture or those lacking fluorescence-associated cell-sorting (FACS) core facilities to quickly adopt these approaches. We have therefore developed an appreciably simplified method (iPS-microglia 2.0) to produce both large numbers and highly purified cultures of human microglia. The resulting cells exhibit RNA transcript profiles that are nearly identical to iPS-microglia generated using our previously published protocol [8], but provide a significantly increased yield at a reduced cost and omit the prior need for a hypoxic incubator and FACS capabilities, making the protocol more readily accessible for a wider variety of labs.

## Results

### The transcriptome of iPS-microglia 2.0 differentiated without hypoxia or cell sorting are almost identical to those generated using our prior approach

**Fig. 1:**
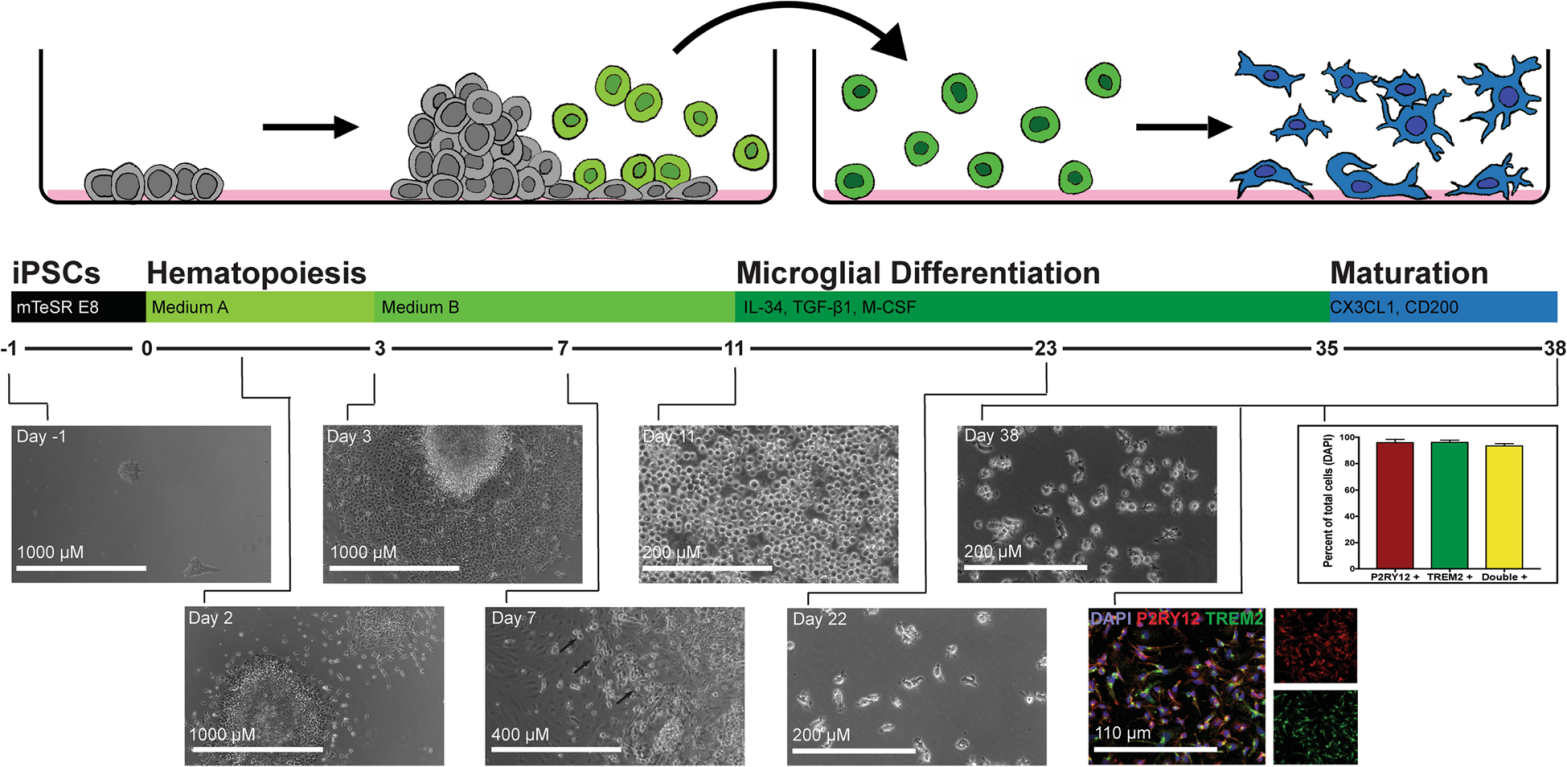
A simplified microglial differentiation protocol can be used to produce large numbers of highly pure human microglia. Schematic showing the process of differentiation from iPSCs through the mesoderm lineage (days 0–3) and further promoting hematopoiesis (days 3–11). Primitive hematopoietic progenitor cells begin to appear on day 7 (black arrows) and by day 11 large numbers of round non-adherent HPCs are observed. Floating HPCs are then transferred into new medium to induce microglial differentiation for 27 days. The last 3 days of microglial differentiation include additional neuronal and astrocytic ligands to further educate microglia toward a brain-like, homeostatic environment. By day 38, large numbers of highly pure microglia that stain positively for both P2RY12 and TREM2 (> 94%) have been produced and are ready for experimentation

Our new differentiation protocol still mimics in vivo microglia ontogeny by first differentiating iPSCs into hematopoietic progenitor cells (HPCs), followed by passage into microglial differentiation medium, and concludes with a final maturation step by adding neural and astrocytic factors, thereby educating the microglia in a brain-like environment while maintaining a pure, homeostatic population of microglia (Fig. 1).

In order to test whether or not an extensively simplified and commercially available hematopoietic stem cell differentiation protocol could be used to generate equivalent homeostatic microglia, we differentiated four independent iPSC lines, and one ESC line (H9) in parallel using our previously published protocol [8] (iPS-microglia) and the simplified iPS-microglia 2.0 protocol described here. Our prior protocol required FACS to isolate CD43^+^ hematopoietic progenitors before transitioning to microglial differentiation medium containing IL-34, TGFβ-1, and M-CSF [14–16]. To determine whether FACS sorting was also necessary for our newer approach, we compared three different sorting methods on the same iPSC background. On day 11 when HPCs were ready to be transitioned into microglial medium, we isolated CD43^+^ HPCs using either FACS, magnetic-activated cell sorting (MACS), or by simply collecting all non-adherent cells. After analyzing transcriptome changes through RNA-sequencing on these samples, we performed unbiased clustering and found that FACS- and MACS-sorted iPS-microglia 2.0 intercluster with unsorted samples demonstrating that these isolation procedures are not necessary for this updated protocol (Additional file 1: Figure S1). Notably, one of the first results we observed with this simplified protocol was that the number of CD43^+^ HPCs produced using this novel commercially available method was substantially increased, while still maintaining the high degree of purity (90–94%) for the HPC marker CD43 (Additional file 2: Figure S2). From one million starting iPS cells, 125 million CD43^+^ cells can be produced, representing an approximately 60-fold increase over our prior method. Following transition to microglial medium, the four lines of unsorted HPCs were further differentiated and matured. At the final day of microglial maturation, iPS-microglia or iPS-microglia 2.0 were harvested for RNA isolation and analyzed via RNA sequencing.

**Fig. 2:**
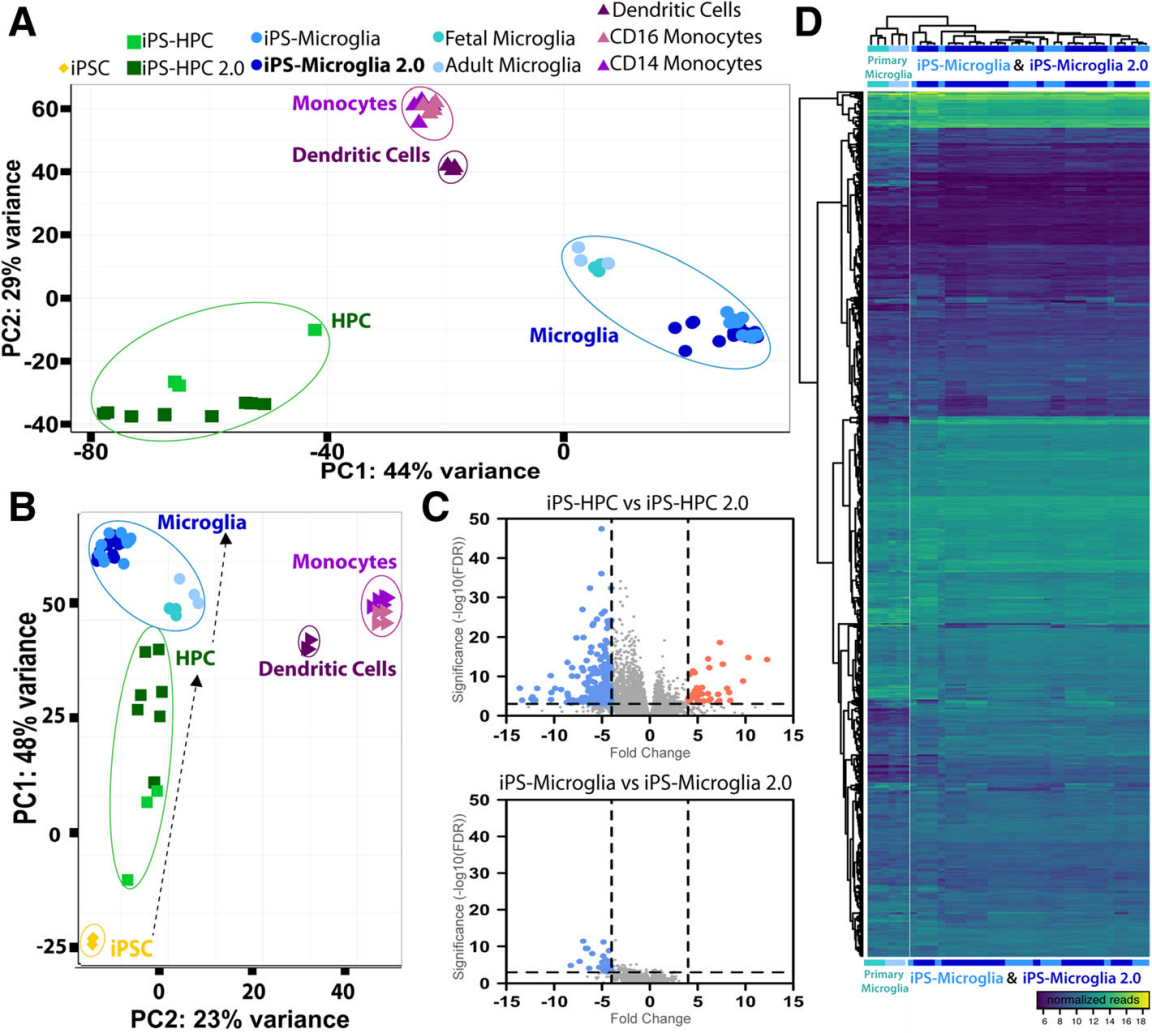
iPS-microglia 2.0 are virtually identical to iPS-microglia generated using a more complex protocol. **a** Principle component analysis demonstrates that iPS-microglia 2.0 (dark blue) and iPS-microglia differentiated using our previously published protocol (blue) exhibit highly equivalent gene expression profiles that cluster closely with cultured human fetal and adult microglia (light blue and teal). Additionally, these cells are distinct from human CD14+ monocytes (purple) and CD16+ inflammatory monocytes (pink), and dendritic cells (maroon). **b** Principal component analysis using a gene list enriched for 882 microglial genes from (Gosselin et al., 2017), further demonstrates the equivalent gene expression between iPS-microglia and fetal and adult microglia. This analysis also highlights the trajectory of differentiation from iPSCs to Microglia and shows the separation between our protocol and monocytic and dendritic cell populations. **c** Volcano plot of differential expression analysis (*p* < 0.001, log2(FC) > 2) between iPS-HPC and iPS-HPC 2.0 samples (top) as well as iPS-microglia and iPS-microglia 2.0 (bottom). Significantly increased or decreased genes are shown in coral or blue respectively. **d** Heatmap using 882 microglial-enriched genes further demonstrates the highly similar gene expression profiles between iPS-microglia and iPS-microglia 2.0 and the close similarity of both cell populations to fetal and adult cultured microglia

**Fig. 3:**
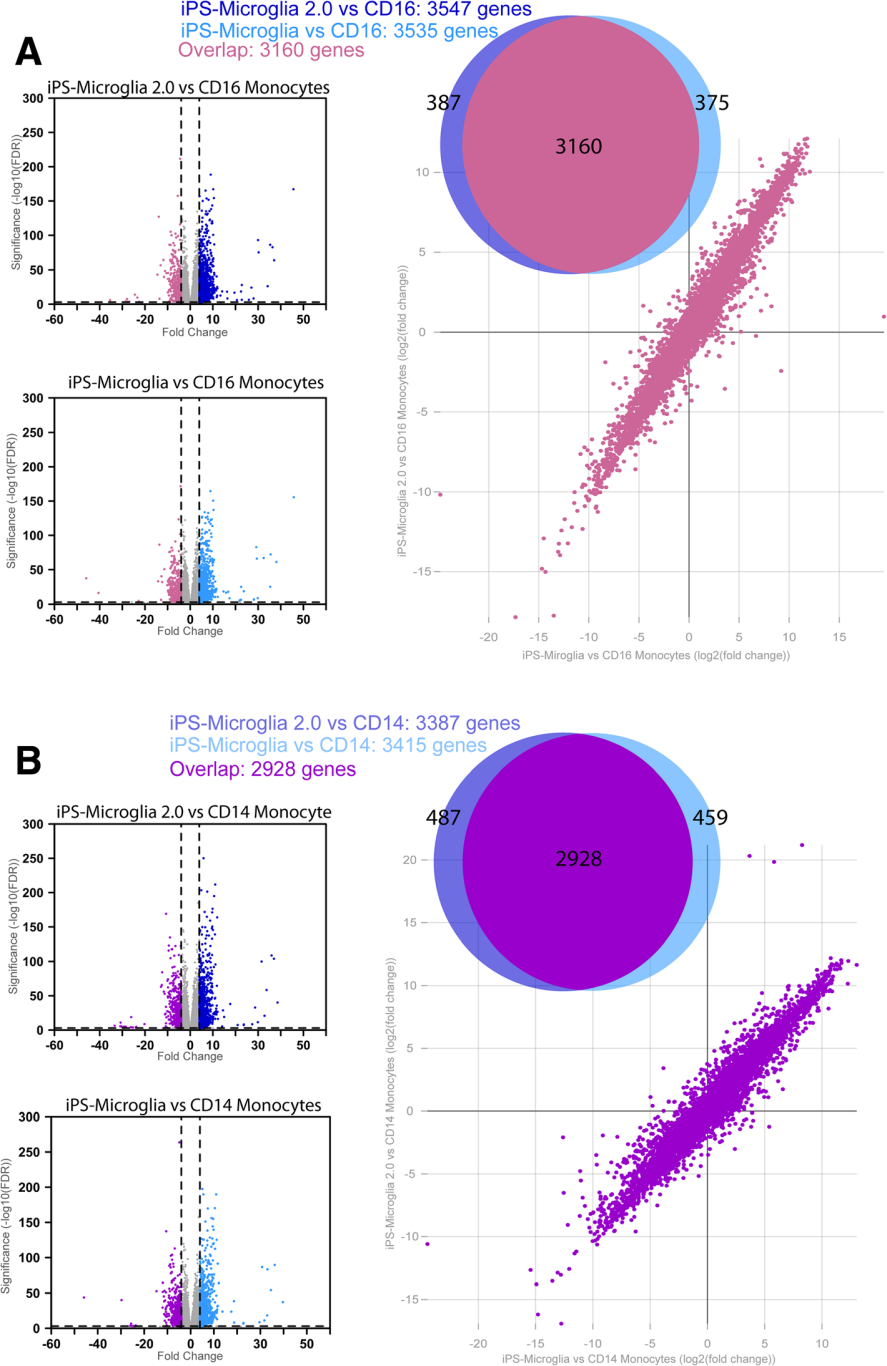
iPS-microglia 2.0 are distinct from CD14^+^ and CD16^+^ monocytes Microglia differentiated using our published protocol are distinct from CD14^+^ monocytes and CD16^+^ inflammatory monocytes. In order to ensure our iPS-microglia 2.0 are similarly distinct from monocytes, differential expression analysis was computed with DEseq2. **a** Volcano plots of differentially expressed genes comparing genes enriched in CD16^+^ monocytes (pink) with genes enriched in iPSmicroglia 2.0 (dark blue) or iPS-microglia (light blue) show many significant differences between monocytes and microglia. Venn diagrams and comparative fold change plots of differentially expressed genes show that the vast majority of differences are identical between iPS-microglia and iPS-microglia 2.0 when compared to CD16^+^ monocytes. Direct comparisons of the fold change expression level (TPM) of every gene are shown in comparative fold change plots which demonstrate the striking similarity of differential expression when iPS-microglia and iPS-microglia 2.0 are each compared to CD16^+^ monocytes. **b** The same is true for comparisons of iPS-microglia and iPS-microglia 2.0 with CD14^+^ monocytes (purple)

At a transcriptome level, our new protocol produced homeostatic human microglia that were virtually identical to microglia generated using our prior methods. Principal component analysis of the full transcriptome explained 73% of variation in all samples within PC1 (44%) and PC2 (29%) and revealed that our new microglia (iPS-microglia 2.0) closely cluster and are interspersed with microglia differentiated using our previously published protocol, yet are highly distinct from human CD14^+^ or CD14/CD16^+^ blood monocytes and dendritic cells (Fig. 2a, b, Fig. 3, Additional file 3: Table S1). Consistent with our previous findings, both iPS-microglia and iPS-microglia 2.0 exhibit very similar gene expression profiles to brain-derived cultured human microglia, although some differences between these groups remain. In order to highlight important microglial and monocyte enriched genes, we performed a secondary principal component analysis with a previously-identified microglial/monocyte focused gene set which revealed a developmental component (PC1, 48% of variance) and again shows interclustering of iPS-microglia 2.0 with microglia generated using our previously published approach (Fig. 2b, d). Importantly, this developmental trajectory remains quite distinct from monocytes and dendritic cells (Fig. 2b).

Interestingly, RNA sequencing analysis of HPCs generated using our previously published approach versus HPC 2.0 revealed some intriguing differences between these two populations. Based on the developmental trajectory shown in Fig. 2b, these data suggest that HPC 2.0 samples are closer to microglia on the primary principle component showing the developmental trajectory than HPCs from our previously described protocol. Indeed HPC 2.0 populations include lower percentages of cells expressing the primitive HPC marker CD235a, although both populations express equivalently high levels of another primitive HPC marker; CD43 which is typically absent in definitive HPCs [17] (Additional file 2: Figure S2, Additional file 4: Table S3). Importantly, although some differences do exist in gene expression between these two HPC populations, very few differences in gene expression persist once HPCs are matured into microglia (Fig. 1, Fig. 2c, d, Additional file 5: Table S2). For example, expression analysis comparing iPS-microglia and iPS-microglia 2.0 revealed only 55 differentially expressed genes. To determine whether these differences occur in a uniform pathway or are indicative of important functional effects that need to be considered, we next used Reactome 2016 gene ontology analysis to examine these 55 genes. This analysis revealed only three significant gene ontology pathways (*Platelet activation, signaling and aggregation_Homo sapiens_R-HAS-76002*, FDR = 0.03665; *Chemokine receptors bind chemokines_Homo sapiens_R0HSA-380,108*, FDR = 0.03665; *Tryptophan catabolism_Homo sapiens_HSA-72,140*, FDR = 0.03665). The chemokine receptor pathway is of course important for microglial function, although this pathway was implicated by only 3 differentially expressed genes: CXCL10, CCL5, and PF4. Thus, we expect that microglial functional activity will be largely equivalent between iPS-microglia and iPS-microglia 2.0.

### Functional validation of iPS-microglia 2.0

**Fig. 4:**
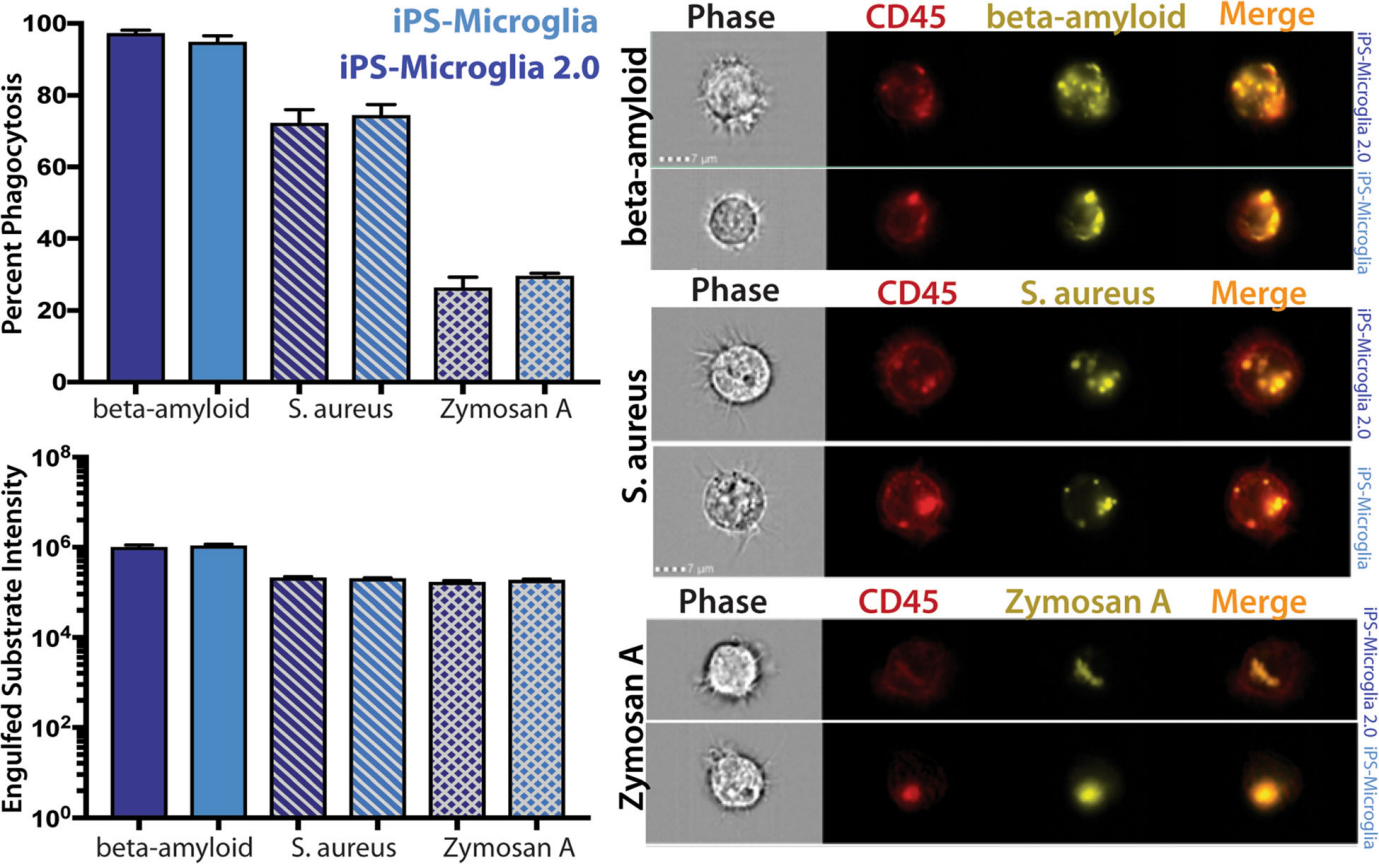
iPS-microglia 2.0 exhibit equivalent substrate-dependent phagocytosis. iPS-microglia and iPS-microglia 2.0 were exposed to fluorescent beta-amyloid fibrils, pHrodo tagged *S. aureus*, or pHrodo tagged Zymosan A bioparticles from *S. cerevisiae*. Quantification of the percent of total cells with positive fluorescent signal and the mean fluorescence intensity of that signal is shown on the left. No significance differences were found between each differentiation type, demonstrating the equivalent functional activity of microglia generated by these two differentiation paradigms. Representative images of phase, CD45 staining, and the fluorescent signal of beta-amyloid (top), *S. aureus* (middle), and Zymosan A (bottom) are shown on the right. One representative image of 10,000 quantified images is shown for iPS-microglia 2.0 (top of each set) and iPS-microglia (bottom of each set)

To determine whether the functional activity of iPS-microglia 2.0 is indeed equivalent to microglia generated using our prior approach, we next compared phagocytic activity of cells generated using both methods [8]. Since the ability of microglia to clear pathogens and extracellular aggregates via phagocytosis is an important aspect of microglial function, we exposed microglia to several different substrates and measured the percentage of cells which phagocytose each substrate using an Amnis Imagestream which combines flow cytometry and high throughput immunofluorescence (Fig. 4). As expected, the levels of phagocytosis differed between the three varying substrates with beta-amyloid fibrils producing the highest response. *S. aureus* bioparticles, a TLR 1,2,6 agonist produced an intermediate degree of phagocytosis and Zymosan, a TLR 2/Dectin 1 agonist from *S. cerevisia* induced the lowest level of phagocytosis (Fig. 4). Importantly, regardless of the differential response to these three phagocytic substrates, iPS-microglia and iPS-microglia 2.0 exhibited identical rates of phagocytosis for each of the substrates, demonstrating that this simplified differentiation method does not alter this important microglial function (Fig. 4).

### iPS microglia 2.0 engraft well into xenotransplantation-compatible MITRG mice

**Fig. 5:**
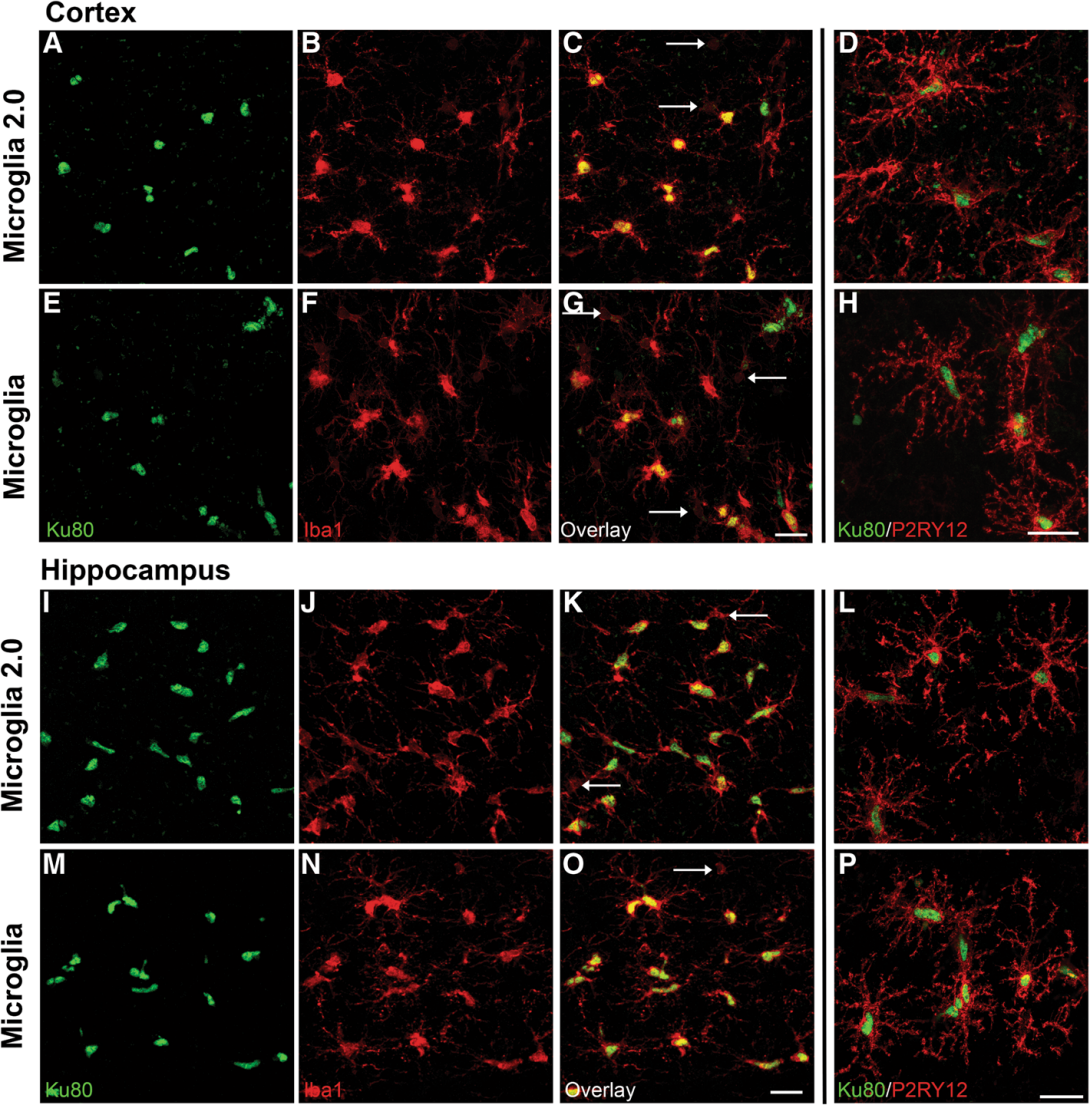
Transplanted iPS-microglia 2.0 display typical microglial markers and morphology comparable to our previously described iPS-microglia. Adult 2 month old MITRG mice were transplanted with (**a-d** & **i-j**, top rows) iPS-microglia 2.0 or (**e-h** & **m-p**, bottom rows) iPS-microglia. Brains were harvested 2 months post-transplant. Representative images of cortical (**a-h**) and hippocampal (**i-p**) transplanted cells demonstrate complex process ramification and typical tiling. Transplanted iPS-microglia and iPS-microglia 2.0 both express Iba-1 (Overlay images **c**, **g**, **k**, & **o**, red) and the microglia specific marker, P2RY12 (Overlay images **d**, **h**, **l**, & **p**, red) and demonstrate human nuclear staining (Ku80, green). Additionally, transplanted human microglia can be seen integrating and tiling with the endogenous mouse microglia population (Arrows indicate Iba1+/Ku80- cells)

We previously demonstrated that iPS-microglia can engraft and ramify, fulfilling characteristic microglia morphology and marker expression in the brains of xenotransplantation-compatible MITRG (Knock-out: Rag2; Il2rg; Knock-in: M-CSFh; IL-3/GM-CSFh; TPOh) mice [8]. Thus, we aimed to further validate the identity of our iPS-microglia 2.0 through intracranial transplantation of iPS-microglia 2.0 into MITRG mice, and to compare this engraftment to equivalently transplanted iPS-microglia that were generated using our previously described differentiation method. In each case, fully mature microglia were transplanted into the hippocampus and overlaying cortex of adult mice which were sacrificed after 2 months for histological examination of morphology and key marker expression. Both iPS-microglia and iPS-microglia 2.0 can be identified within the mouse brain via expression of the human-specific nuclear marker, Ku80 (Fig. 5, green). Importantly, regardless of the differentiation method, transplanted human microglia display typical microglial morphology, extending complex branching processes. Both iPS-microglia and iPS-microglia 2.0 also express the microglial/monocyte marker Iba1 (Fig. 5, Overlay images C, G, K, & O, red) and the homeostatic microglial marker P2RY12 (Fig. 5 Overlay images, D, H, L, & P, red) in both cortex and hippocampus, indicating that these cells engraft well and remain homeostatic. Transplanted iPS-microglia 2.0 also exhibit the tiling and distinct niches typical of in vivo microglia, and can be seen interspersed with the endogenous population of mouse microglia (Fig. 5, arrows indicate Iba1^+^/Ku80^−^ mouse cells). Taken together, these findings further demonstrate that iPS-microglia 2.0 are equivalent to microglia generated using our previously published protocol and can be readily transplanted into MITRG mice to enable in vivo studies of human microglia*.*

### Small molecule activation of TGFβ signaling produces microglia-like cells that are similar, but transcriptionally distinct from iPS-microglia 2.0

TGFβ1 is a crucial astrocyte-derived cytokine that promotes microglial homeostasis [15, 18]. TGFβ1 signaling results in phosphorylation of smad2/3 and ultimately up-regulates expression of CX3CR1, an important receptor for microglial function and survival [19, 20]. Indeed, removal of either TGFβ1 or CX3CR1 greatly decreases microglia populations in murine models [15, 20]. As we have previously shown, removal of TGFβ1 from iPS-microglia for even 24 h also results in dramatic changes in the microglial transcriptome, including down-regulation of homeostatic signatures [8].

In order to increase cost-efficiency during iPS-microglia 2.0 differentiation, we attempted to replace recombinant TGFβ1 with Inducer of Definitive Endoderm 1 or 2 (IDE1, IDE2). As its name suggests, IDE has been used to differentiate iPS cells into definitive endoderm through activation of TGFβ signaling [21]. More specifically, IDE1/2 have been shown to induce phosphorylation of the downstream TGFβ signaling molecule smad2 [21]. This led us to hypothesize that IDE1 or IDE2 could induce expression of microglial genes in an equivalent fashion to recombinant TGFβ if added after mesoderm formation and hematopoiesis.

**Fig. 6:**
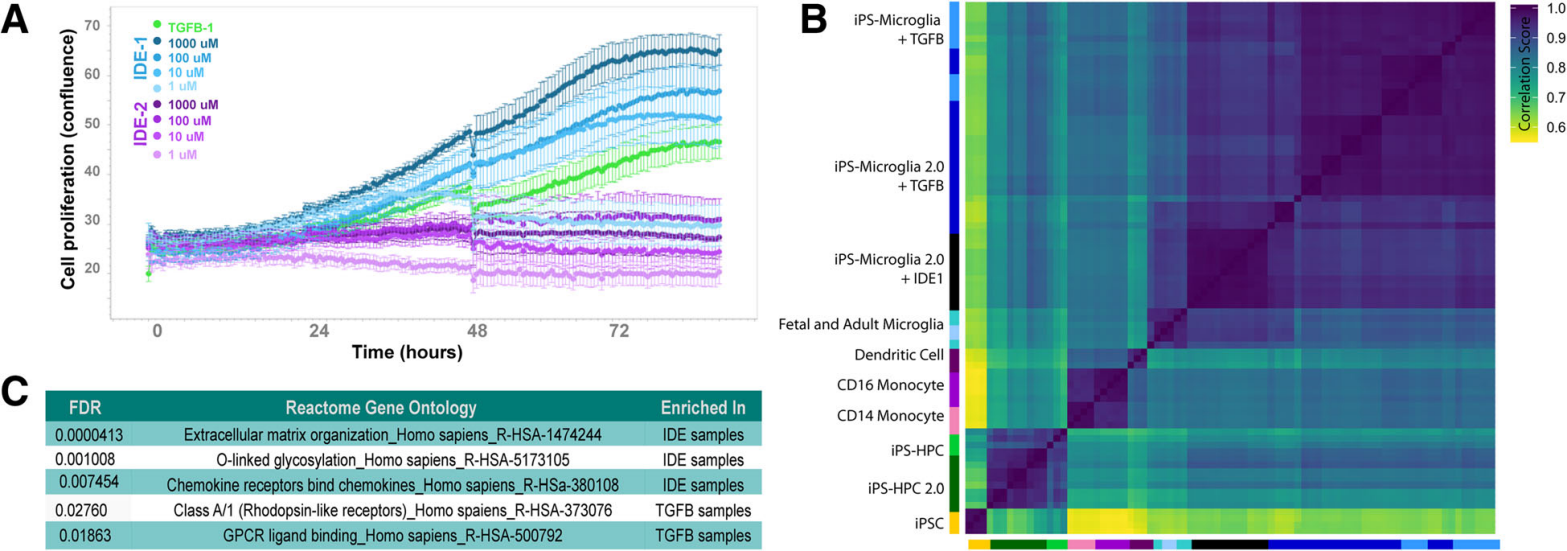
The small molecule compound IDE1 can be used in place of TGFβ-1 to produce iPS-microglia. IDE1 and IDE2 were added to microglia cultures in place of TGFβ-1 at the indicated concentrations. **a** Growth curves from the first 3.5 days of microglial differentiation show that IDE2 is insufficient to allow proliferation of these cells. In contrast, IDE1 (blue) at lower concentrations shows similar growth kinetics to TGFβ control cells (green). **b** Correlation matrix displaying all samples analyzed in this manuscript shows cells differentiated with IDE1 cluster closely with iPS-microglia 2.0 and are actually more similar to fetal and adult microglia than TGFβ control microglia. **c** Gene ontology analysis using the Reactome database displays differences between IDE1 treated cells and TGFβ (FDR < 0.001, FC > 2). Enrichment in IDE samples reflects an increased expression of genes within this GO family in IDE1 treated cells

To determine whether IDE1 or IDE2 could replace recombinant TGFβ in our differentiation protocol, HPCs harvested on day 10 were placed into microglia differentiation media and varying concentrations of IDE1 or IDE2 (1 μM, 10 μM, 100 μM, 1000 μM) were added in place of TGFβ (Fig. 6a). During the first 4 days of microglia differentiation we used an Incucyte live imaging system to examine the growth kinetics of each group to provide an initial assessment of the effects of varying IDE concentrations. Surprisingly, this analysis demonstrated that IDE2, regardless of concentration, impaired normal microglial proliferation and thus was not studied further. In contrast, IDE1 was able to mimic the typical growth kinetics observed in control cells differentiated in parallel and maintained in normal TGFβ-containing medium. Because the control TGFβ microglial growth curve fell between the 10 μM and 1 μM IDE1 curves, we next adjusted the IDE1 concentrations to include a 5 μM dose. In addition, two higher concentrations of IDE1 (50 μM, and 500 μM) were also included as the growth kinetic measurements suggested that these concentrations might further increase the yield of microglia.

All groups were differentiated in parallel for the complete 38 day paradigm before RNA-sequencing was performed. To compare these new samples with our iPS-microglia, fetal and adult microglia, and other cell types, we generated a correlation matrix (Fig. 6b) which demonstrated that IDE1 iPS-microglia remain distinct from monocytes and dendritic cells and cluster closely with our other iPS-microglia. IDE-treated cells also exhibited strong expression of key microglial genes including CSF1R, P2YR12, TREM2, OLFML3, HEXB, and C1Q (Additional file 6: Table S4). Based on the hierarchical clustering within our correlation matrix (Fig. 6b), we find that microglia differentiated in IDE1 have transcriptomic profiles that are more similar to primary cultured microglia (fetal and adult microglia). One possible explanation for this finding is that IDE1 may have greater stability within the culture media than TGFβ1 and thus provide a more uniform and consistent activation of TGFβ signaling pathways. Alternatively, IDE1 could potentially provide increased specificity by only targeting smad2 signaling. To further understand the comparative effects of IDE1 versus TGFβ1 we further compared these groups of iPS-microglia using DEseq2 to highlight any differentially expressed genes (Additional file 6: Table S4). Interestingly, gene ontology analysis of these differentially expressed genes revealed only five pathways that were significantly enriched between these two populations of microglia (Fig. 6c), again showing their strong similarity. Taken together these data suggest that IDE1 can indeed be used in place of TGFβ1, although researchers should also consider these differentially altered pathways and genes in there decision whether or not to use this further modified approach.

## Discussion

In recent years, the importance of microglia in brain development, homeostasis, and disease has become increasingly clear. Because microglia have been implicated in many neurological diseases and injuries including neurodegeneration, traumatic brain injury, and developmental disorders, several groups have developed methods to try to make these cells more accessible for neurological research. Until recently, microglia could only be studied through brain biopsies, postmortem analysis, or in animal models. Although mouse models of neuroinflammation have been extremely useful in uncovering important new findings, many differences exist between human and murine microglia. For example, one recent study identified several co-regulated myeloid gene expression modules that occur in human AD, but do not occur in AD mouse models [22]. Likewise, many differences exist between the murine and human complement system that is closely linked to neurodegenerative diseases including AD [6]. At least two microglial-expressed AD risk genes, CR1 and MS4A4A, have no murine ortholog, further highlighting the challenges of studying the role of microglia in human disease using mouse models alone.

To study human microglia, some highly skilled groups have turned to human biopsy material. Working closely with neurosurgeons, these researchers have developed methods to isolate human microglia from brain tissue removed during a surgical resection of a brain tumor or intractable epileptic foci [5, 7, 23]. Using this approach, researchers have uncovered exciting data and greatly advanced our understanding of the human microglial transcriptome. However, epileptic foci and tumor tissue induce neuroinflammation and despite best efforts to avoid isolating microglia from ‘diseased-effected tissue’ it is likely that microglia isolated from these patients exhibit considerable variation and alterations in activation state [24, 25].

Another strategy for studying human microglia involves the isolation of microglia or their nuclei from postmortem brain tissue. Using this approach, researchers have uncovered important age-related differences in the human microglial transcriptome [26]. Still, it remains unclear whether the agonal state that precedes death, inflammatory co-morbidities, or post-mortem delay might influence microglial gene expression. In the case of Alzheimer’s disease, most patients die from an accompanying infectious disease such as Pneumonia [27, 28]. Interestingly, animal models of Pneumonia exhibit significant changes in brain microglial activation state [29, 30]. Thus, it is likely that this and other common infectious co-morbidities can complicate the interpretation and analysis of postmortem-isolated human microglia.

Given the considerable challenges with isolation and study of postmortem or biopsied human microglia, several groups, including our own, developed protocols which utilize the power of stem cells to produce human microglia in vitro [8]–[13]*.* These methods have begun to enable more detailed mechanistic studies of human microglia by allowing controlled experimental treatments, drug testing, and genetic manipulation. However, the currently existing protocols are relatively complicated and can be challenging to adopt, especially for groups with little prior stem cell experience. Thus, to address this challenge we developed and validated the greatly simplified and refined method presented here. In comparing this new method to our previously published differentiation protocol, we confirm that iPS-microglia 2.0 show highly similar RNA transcript profiles to iPS-microglia as well as primary fetal and adult microglia. In addition, iPS-microglia 2.0 remain distinct from blood monocytes and importantly display largely the same differentially expressed genes between microglia and monocytes as our previously published iPS-microglia.

To further investigate and characterize iPS-microglia 2.0 we functionally validated these cells by examining phagocytosis of three different substrates; *Staphylococcus aureus*, Zymosan A, and fibrillar beta-amyloid. While each substrate exhibited differential degrees of phagocytosis, these levels were equivalent between our previously described iPS-microglia and iPS-microglia 2.0. Lastly, to determine whether iPS-microglia 2.0 can also be used for in vivo studies, we transplanted microglia derived via both methods into xenotransplantation-compatible MITRG mice, confirming that engraftment, in vivo morphology, and marker expression was equivalent between iPS-microglia and iPS-microglia 2.0. Taken together, these functional and in vivo experiments further support the conclusion that microglia generated via these two methods are virtually identical.

In addition, we tested IDE1 as a small molecule agonist of TGFβ signaling cascades. To this end, we confirmed that substitution of TGFβ1 with IDE1 produced cells that are similar to iPS-microglia 2.0, and additionally highly similar to adult and fetal primary microglia. We have provided differential gene expression analysis to highlight the important differences between IDE- and TGFβ1-treated iPS-microglia 2.0, which researchers should consider when deciding whether to use TGFβ or cost-saving IDE1 for iPS-microglia generation.

## Conclusions

In summary, we provide detailed methods and validation of a greatly simplified protocol to produce significantly increased numbers of pure human microglia. The RNA-sequencing, functional validation, and transplantation studies presented here clearly demonstrate that highly pure populations of human iPS-microglia can be generated via this greatly simplified protocol. We anticipate that this streamlined and highly reproducible protocol will enable many more interested researchers to now study human microglia, leading to further breakthroughs in this field.

## Methods

Ethics Statement: All experiments were carried out according to human stem cell (hSCRO) and animal use (IACUC) protocols that were approved by the University of California, Irvine.

Find the complete catalog of materials and catalog numbers in Additional file 7: Table S5.

### Simplified differentiation of iPSCs to HPCs

Improved and simplified differentiation of iPSCs to CD43^+^ primitive hematopoietic progenitor cells (HPCs) is achieved using Stem Cell Technologies STEMdiff™ Hematopoietic Kit (Catalog # 05310). On day − 1, feeder-free iPSCs that have been expanded in TeSR-E8 media are passaged with ReLeaSR (STEMCELL technologies) into mTeSR E8 medium with 0.5 μM Thiazovivin onto matrigel coated (1 mg/mL) 6-well plates (Corning Costar). Small aggregates of ~ 100 cells each are plated at 10–20 aggregates per cm^2^. The initial plating density is critical as higher density impairs mesoderm differentiation and lower density decreases yield. Thus one can plate iPSCs at 2–3 different densities and select the wells on day 0 that have optimal density to proceed with. When approximately two 100 cell colonies per cm^2^ have been achieved, replace TeSR-E8 medium with medium A (Basal medium plus Supplement A at 1:200 dilution, 2 mL per well of a 6-well). On day 2 (48 h after original media change), do not fully change media, but rather replace 50% medium A, 1 mL per well of a 6-well. On day 3, carefully remove all media by tilting the plate to one side and aspirating from the edge. Then add 2 mL/well medium B (Basal medium plus supplement B at 1:200). Without removing media, supplement with 1 mL/well of medium B on days 5, 7, 9. On day 10 and again on day and 12, non-adherent cells may be collected. To maintain purity, do not wash cells off the well, but merely remove medium with non-adherent cells carefully and centrifuge 300 x G 5 min. After centrifugation, replace conditioned medium back to each well and add 1 mL fresh medium B if further collection on day 12 will be completed.

FACS analysis has confirmed that these non-adherent cells represent highly pure populations (> 93%) of CD43^+^ hematopoietic progenitor cells (Additional file 2: Figure S2). Importantly, simply collecting the floating cells is all that is required to isolate large numbers of highly purified CD43^+^ cells. No FACS or MACS isolation is required as identical microglia are produced using any of these three methods (Additional file 1: Figure S1). However, because the cells are not being sorted for purity, the collection of non-adherent cells must be carefully completed. Do not spray medium over adherent cells to wash as this will loosen cells which are not CD43^+^ and decrease culture purity.

At this point, HPCs may be frozen at 2–4 million cells per mL in BamBanker (Wako). If frozen, HPCs should be thawed directly into microglial differentiation medium with cytokines (below) and plated onto Matrigel-coated plates at 10,000 cells per cm^2^. We typically find that viability post-thaw is between 70-95%, with improved viability when greater densities of HPCs are thawed together.

### Updated differentiation of CD43^+^ HPCs to iPS-microglia 2.0

Volumes specified for 35 mm well (1 well of a 6-well plate).

On day 0 of iPS-microglia differentiation, plate HPCs at 10,000 cells per cm^2^ onto 1 mg/mL Matrigel-coated plates (100,000 per 35 mm well). Plate cells into iPS-microglia medium at 2 mL per 35 mm well: DMEM/F12, 2X insulin-transferrin-selenite, 2X B27, 0.5X N2, 1X glutamax, 1X non-essential amino acids, 400 μM monothioglycerol, 5 μg/mL insulin. Immediately before use, microglial medium should be supplemented with 100 ng/mL IL-34, 50 ng/mL TGFβ1, and 25 ng/mL M-CSF (Peprotech) taken from single-use frozen aliquots (important: do not freeze/thaw these cytokines as it will significantly impair differentiation and yield as well as induce activation. It is crucial to thaw cytokines immediately before use). Throughout the differentiation of HPCs to microglia, these cells will predominantly grow non-adherently. On days 2, 4, 6, 8, and 10, add 1 mL fresh media plus freshly thawed tri-cytokine cocktail. Cytokines are diluted to the concentrations listed above before adding to conditioned medium. Do not fully remove media during the microglial differentiation as the cells secrete paracrine cytokine signals and will not properly differentiate upon removal of those. On day 12, collect 6 mL media from each 35 mm well leaving 1 mL conditioned medium on the plate. Centrifuge non-adherent cells in removed medium for 5 min at 300 x G. Aspirate medium and resuspend non-adherent cells in 1 mL fresh medium plus tri-cytokine cocktail per 35 mm well and add back to the same well which contains the 1 mL conditioned medium. Continue to supplement media (1 mL) on days 14, 16, 18, 20, 22, and 24. On day 25, centrifuge cells leaving 1 mL conditioned media per 35 mm well as on day 12. On day 25, cells should be resuspended in microglia media plus 100 ng/mL IL-34, 50 ng/mL TGFβ1, 25 ng/mL M-CSF, 100 ng/mL CD200 and 100 ng/mL CX3CL1 to further mature microglia and ensure homeostasis. On day 27, feed cells with microglia media with five cytokine cocktail (1 mL per well). On day 28 cells collected for RNA sequencing or use for transplantation or functional assays. If necessary, cells can be maintained for 1–2 additional weeks via media supplementation as above, although longer-term culture is not advised.

### Isolating RNA

Total RNA was isolated from cells using RNeasy Mini kit (Qiagen). Approximately 1 million iPS-microglia cells were lysed in RLT buffer and RNA was isolated per manufacturer’s instructions with DNAse treatment (10 min) and increased spin times to maximize yield (16,000 x G for 1.5 min). RNA integrity was measured using the Bioanalyzer Agilent 2100. All libraries were prepared from samples with RNA integrity values ≥9.7. 500 ng RNA per sample was used to create RNA-seq libraries through the Illumina TruSeq mRNA stranded protocol. Each sample was then sequenced in the Illumina HiSeq 4000 platform.

### RNA sequencing analysis

RNA sequencing read integrity was verified using FastQC. BBDuk was used to trim adapters and filter out poor quality reads [31]. Reads were aligned to the GRCh.38.12 human transcriptome using Kallisto [32]. Lowly expressed genes (expression count summed over all samples < 10) were removed before differential expression analysis. Differential Expression of TPM was calculated using DESeq2 [33]. An FDR cutoff of 0.001 and fold change of at least 2 was used to determine differentially expressed genes (Additional file 3: Table S1, Additional file 4: Table S2, Additional file 5: Table S3, Additional file 6: Table S4). Visualizations were constructed in part using R in addition to the Genialis visual informatics platform (app.genialis.com) [34]. Gene ontology analysis was performed using EnrichR.

### Phagocytosis assay

Phagocytic activity of iPS-microglia was examined using the the Amnis Imagestream (Millipore) to combine immunofluorescence and flow cytometry. iPS-microglia or iPS-microglia 2.0 were treated with either 1 μg/mL pHrodo tagged zymosan A beads, 20 μg/mL *S. aureus*, or 2 μg/mL fluorescent beta-amyloid (Anaspec). After allowing 1 h at 37 degrees for phagocytosis, microglia were resuspended in cold FACS buffer (DPBS, 1% BSA, 0.5 mM EDTA) and stained for 30 min at 4 degrees with 1:100 anti-CD45 (Biolegend, clone HI30) and Zombie-violet live/dead stain. 10,000 events were captured for each sample which were gated for in focus, live cells before analysis. IDEAS software was used to generate masks of internalized signal (substrate within CD45) and percent of cells with internalized substrates were calculated as well as mean fluorescent intensity which remained constant for each cell type.

### Animals

All animal procedures were conducted in accordance with the guidelines set forth by the National Institutes of Health and the University of California, Irvine Institutional Animal Care and Use Committee. The MITRG mouse was purchased from Jackson Laboratories (stock #017711); briefly, this strain was developed on a BALB/c background containing two knockouts alleles: Rag2^−^ (Rag2^tm1.1Flv^) and il2γc^−^ (Il2rg^tm1.1Flv^); and three humanized knock-in alleles: hCSF-1 (Csf1^tm1(CSF1)Flv^), h-IL-3/GM-CSF (Csf2/Il3^tm1.1(CSF2,IL3)Flv^), and hTPO (Thpo^tm1.1(TPO)Flv^). All mice were age and sex matched and group housed on a 12 h/12 h light/dark cycle with food and water ad libitum.

### Adult intracranial transplants

All mouse surgeries and use were performed in strict accordance with approved NIH and AALAC-certified institutional guidelines. Direct intracranial injections of iPS-microglia into the cortex and hippocampus were performed on adult MITRG mice. Briefly, adult mice (2–3 months old) were anesthetized under continuous isoflurane and secured to a stereotaxic frame (Kopf). Using a 30-guage needle affixed to a 10 μL Hamilton syringe, mice received 2 μL of mature iPS-microglia suspended in sterile 1X DPBS at 50,000 cells/μL at each injection site. Transplantation was conducted bilaterally in the cortex and hippocampus at the following coordinates relative to bregma: anteroposterior, − 2.06 mm; dorsoventral, − 1.75 mm (hippocampus), − 0.95 mm; mediolateral, ±1.75 mm. Cells were injected at a rate of 50,000/30s with 4 min in between injections. The needle was cleaned with consecutive washes of PBS, 70% (vol/vol) ethanol, and PBS in between hemispheres and animals. Animals were allowed to recover on heating pads before being placed in their home cages and received 2 mg/mL Acetaminophen (Mapap) diluted in water for five days. Animals were perfused 2 months following surgery with 1X PBS followed by 4% paraformaldehyde, entire brains were removed for immunohistochemistry and confocal microscopy.

### Immunohistochemistry and confocal microscopy

Fixed half brains were first cryoprotected in a 30% sucrose and 0.05% NaN_3_ solution in 1X PBS for 72 h. Tissue was then sectioned into 40 μm thick slices on a freezing microtome (Leica SM 2010R), and stored in 0.05% NaN_3_ solution in 1X PBS as free floating wells. For staining, tissue was blocked for 1 h in 1X PBS, 0.2% Triton X-100, and 10% goat serum. Immediately following blocking, sections were placed in primary antibodies diluted in 1X PBS and 1% goat serum and incubated overnight on a shaker at 4 °C. Sections were labeled with combinations of anti-Ku80 (1:250; Abcam ab79220), anti-Iba1 (1:200; Wako 019–19,741), anti-P2RY12 (1:200; Sigma HPA014518) and mounted with DAPI Fluoromount (SouthernBiotech). Immunofluorescent sections were then visualized and captured using an Olympus FX1200 confocal microscope. Images represent confocal Z-stack taken with equivalent laser and detection settings.

## Additional files

Additional file 1: **Figure S1.** FACS and MACS sorted HPCs differentiate into equivalent microglia as unsorted HPCs. Hierarchical clustering of all iPSmicroglia 2.0 samples show that MACS (fuchsia) and FACS (purple) sorting at the HPC stage has no effect on the final differentiated microglia as these samples intercluster with iPS-microglia 2.0 produced from floating unsorted HPCs (blue). (TIF 11571 kb)

Additional file 2: **Figure S2.** Both HPC and HPC 2.0 methods produce cells with consistently high expression of the primitive HPC marker CD43. (Top) Quantification of flow cytometry analysis from four independent iPSC lines per method (*n* = 3 wells/line) reveal a similarly high proportion of cells (> 90%) that express the primitive HPC marker CD43 following differentiation with either our previous or currently described approach. In contrast, two other primitive HPC markers, CD41 and CD235a, exhibit relatively low and heterogeneous expression within this CD43+ population (Middle). Representative FACS plots demonstrating typical CD43+ histograms (pre gated for live, single cells). Gates were drawn based on FMO (fluorescence minus one) controls. (Bottom) Heatmap from RNA sequencing of iPS-HPC samples shows similar gene expression levels for CD43, CD41, and CD235a. (TIF 45670 kb)

Additional file 3: **Table S1.** Significantly changed genes between iPS-microglia, iPS-microglia 2.0, CD14 monocytes, and CD16 monocytes. (XLSX 1562 kb)

Additional file 4: **Table S3.** Differential expression analysis of iPS-HPC versus iPS-HPC2.0. (XLSX 11 kb)

Additional file 5: **Table S2.** Differential expression analysis of iPS-microglia versus iPS-microglia2.0. (XLSX 80 kb)

Additional file 6: **Table S4.** Differential expression analysis comparing IDE-treated microglia to TGFB control microglia or fetal and adult cultured microglia. (XLSX 11597 kb)

Additional file 7: **Table S5.** Catelog numbers for all materials used in this manuscript. (XLSX 45686 kb)

## Abbreviations

AD: Alzheimer’s disease
C1Q: Complement component 1q
CD: Cluster of differentiation
CR1: Complement receptor 1
CRISPR: Clustered regularly interspersed short palindromic repeats
CX3CL1: Fractalkine
CX3CR1: C-X3-C motif chemokine receptor 2
DPBS: Dulbecco’s phosphate buffered saline
FACS: Fluorescence-associated cell-sorting
FDR: False discovery rate
GM-CSF: Granulocyte-macrophage colony-stimulating factor
HC1: Histone H1-like
HEXB: Hexosaminidase subunit beta
HPC: Hematopoietic progenitor cell
IBA1: Ionized calcium binding adaptor molecule 1
IDE: Inducer of definitive endoderm
IL2rg: Interleukin 2 receptor subunit gamma
IL-34: Interleukin 34
iPS/iPSCs: Induced pluripotent stem cells
MACS: Magnetic-associated cell-sorting
MCSF/CSF1: Macrophage colony-stimulating factor
MITRG: Microglia transplant compatible mouse strain see https://www.jax.org/strain/017711
mRNA: messenger RNA
MS4A4A: Membrane-spanning 4-domains subfamily A member 4A
OLFML3: Olfactomedin like 3
P2RY12: Purinergic receptor P2Y12
PBS: Phosphate buffered saline
PC: Principal component
pHrodo: pH sensitive dyes
RABC3: Ras related protein Rab3c
Rag2: Recombination activating 2
RNA: Ribonucleic acid
TGFβ: Transforming growth factor β
TPO: Thrombopoietin
TREM2: Triggering receptor expressed on myeloid cells 2
