# Supplementary figures and images for: Development and validation of a simplified method to generate human microglia from pluripotent stem cells
Source: Mol Neurodegener. 2018 Dec 22;13:67. doi: 10.1186/s13024-018-0297-x (PMC6303871; doi:10.1186/s13024-018-0297-x)

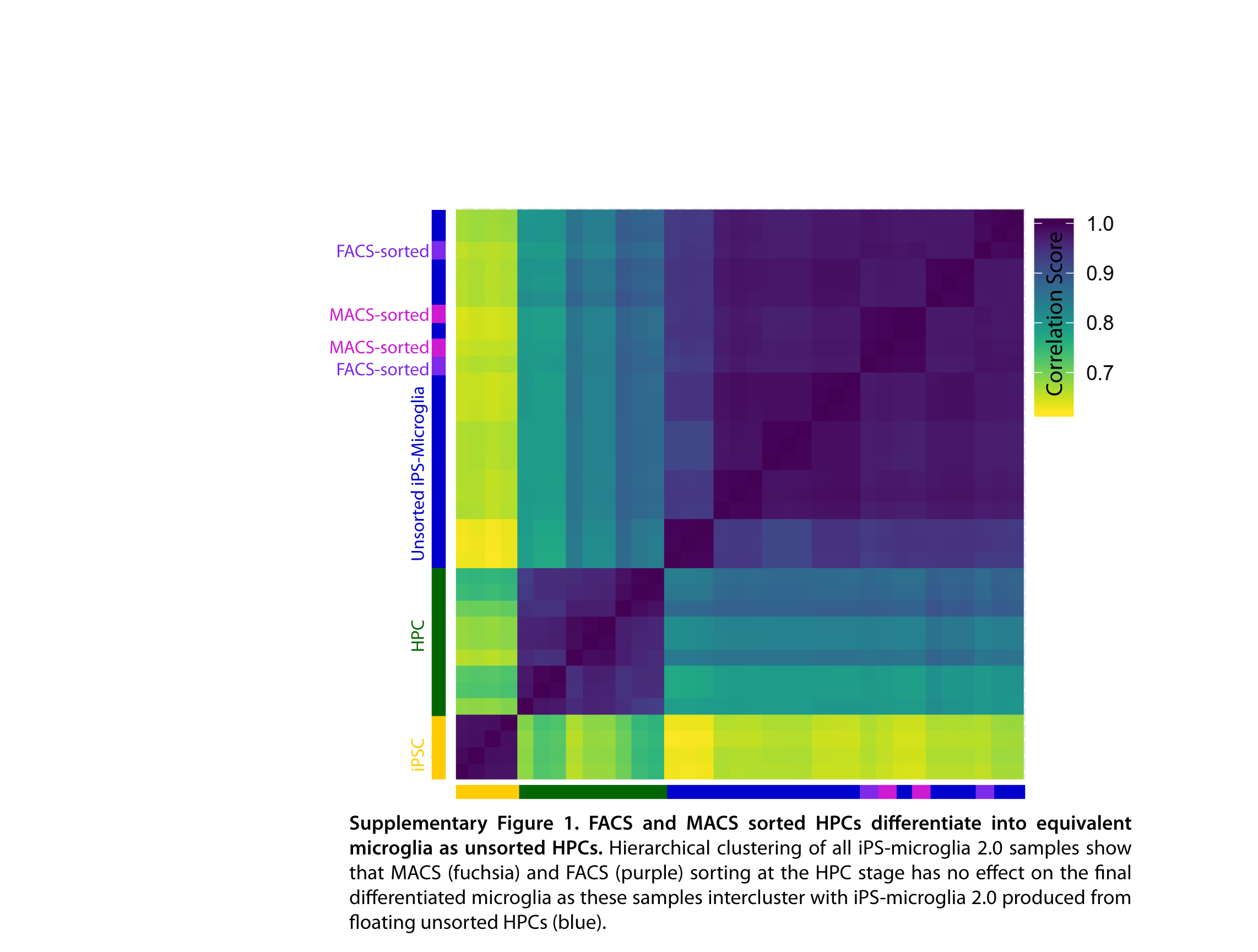

Supplement: Supplementary file 1 — Figure S1. FACS and MACS sorted HPCs differentiate into equivalent microglia as unsorted HPCs. Hierarchical clustering of all iPSmicroglia 2.0 samples show that MACS (fuchsia) and FACS (purple) sorting at the HPC stage has no effect on the final differentiated microglia as these samples intercluster with iPS-microglia 2.0 produced from floating unsorted HPCs (blue). (TIF 11571 kb) [file 13024_2018_297_MOESM1_ESM.tif]

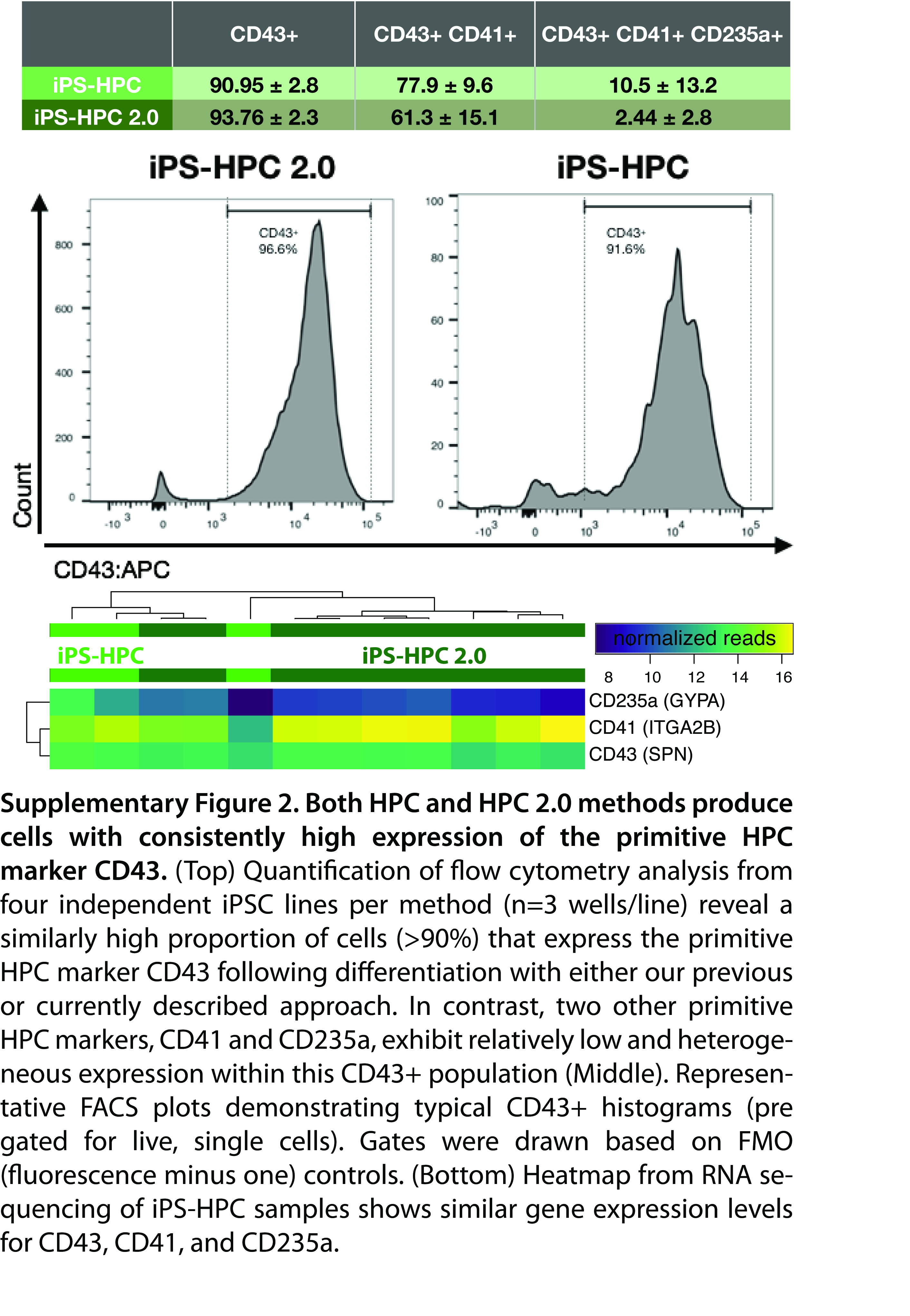

Supplement: Supplementary file 2 — Figure S2. Both HPC and HPC 2.0 methods produce cells with consistently high expression of the primitive HPC marker CD43. (Top) Quantification of flow cytometry analysis from four independent iPSC lines per method (n = 3 wells/line) reveal a similarly high proportion of cells (> 90%) that express the primitive HPC marker CD43 following differentiation with either our previous or currently described approach. In contrast, two other primitive HPC markers, CD41 and CD235a, exhibit relatively low and heterogeneous expression within this CD43+ population (Middle). Representative FACS plots demonstrating typical CD43+ histograms (pre gated for live, single cells). Gates were drawn based on FMO (fluorescence minus one) controls. (Bottom) Heatmap from RNA sequencing of iPS-HPC samples shows similar gene expression levels for CD43, CD41, and CD235a. (TIF 45670 kb) [file 13024_2018_297_MOESM2_ESM.tif]
